# Acoustic Multi-Parameter Early Warning Method for Transformer DC Bias State

**DOI:** 10.3390/s22082906

**Published:** 2022-04-10

**Authors:** Yuhao Zhou, Bowen Wang

**Affiliations:** 1 International Education Institute, North China Electric Power University (Baoding), Baoding 071003, China; zhouyuhaoncepu@163.com; 2 Hebei Provincial Key Laboratory of Power Transmission Equipment Security Defense, North China Electric Power University (Baoding), Baoding 071003, China

**Keywords:** transformer, acoustic detection, DC bias, data statistics

## Abstract

The acoustic signal in the operation of a power transformer contains a lot of transformer operation state information, which is of great significance to the detection of DC bias state. In this paper, three typical parameters used for DC bias state detection are selected by comparing the acoustic variation of a 500 kV Jingting transformer substation No. 2 transformer with that of the core model built in the laboratory; then, acoustic samples of the 162 EHV normal state transformers are collected, and the distribution regularity of three typical parameters in normal state is given. Finally, according to the distribution regularity, clear warning threshold of typical parameters are given, and the DC bias cases from the 500 kV Jingting transformer substation are used to verify the effectiveness of the threshold.

## 1. Introduction

With EHV transmission projects widely used, the number and voltage level of DC transmission lines are growing, and the DC magnetic bias problem caused by AC/DC hybrid transmission is becoming increasingly serious [1,2,3,4]. Transformer DC magnetic bias monitoring is usually carried out by measuring grounding current [5,6,7,8,9], which requires electromagnetic coupling and has weak safety. As a mechanical wave detection method, acoustic signal detection can realize the mechanical state detection of equipment without electromagnetic coupling. This method has been widely used in saturable reactor monitoring [10,11], motor monitoring [12,13], and other fields. During the operation of the transformer, the core, winding, and other structures will vibrate and produce mechanical waves. The generated acoustic signal contains a lot of equipment status information, which can realize transformer DC bias state online monitoring [14,15,16,17].

In recent years, the acoustics or vibrations of a transformer have been analyzed from the perspective of mechanism explanation and experimental simulation [18,19,20,21,22,23]. Secic summarized works related to the acoustic condition assessment of power transformers [24]. In terms of transformer acoustic signal parameters, Bartoletti used the total harmonic distortion (THD) to identify the health condition of the transformer winding [25]. Hong presented frequency complexity (FC), determinism (DET), energy difference ratio (EDR), and main principal contribution (MPC) to diagnose winding health [26]. Belén García presented a transformer tank vibration estimate model [27], and experimental verification was carried out [28]. Hong Z. proposed a winding vibration model coupled with electromagnetic force analysis [29], and the model was helpful to predict the mechanical faults of transformer windings. Zhang F. studied the acoustic transmission model of a transformer and proved that the vibration of the oil tank is mainly affected by the oil-borne transmission [29]. The above theoretical research has carried out a separate analysis of the possible frequency phenomena in various states of the transformer, which provides a lot of analysis and judgment basis for the analysis of sound and vibration signals, which has important reference value. However, the current research only uses the data of the laboratory simulation platform, or only uses the data of the actual transformer, and different objects lead to different conclusions. It is rare to compare and analyze the voiceprint characteristics of the actual transformer and the laboratory simulation platform in the literature. This work is conducive to finding out the useful common acoustic parameters. In addition, the early warning threshold is also fuzzy for various acoustic parameter indicators in practical application, which needs to be further clarified.

In order to obtain reliable acoustic parameters of DC bias state and its definite early warning threshold, this paper first carries out the DC bias state acoustic signal collection on the 500 kV transformer (from Jingting Substation in Anhui Province) and the transformer model (built in the laboratory). Secondly, two sets of acoustic signal samples parameters are calculated and compared, and three typical parameters that are beneficial to the detection of the DC bias state are screened out. Then, the typical parameters of 162 normal state single-phase transformers in Hebei Power Grid are statistically analyzed, which provide data support for the delineation of early warning threshold and use real DC bias case to verify the effectiveness of the threshold application.

## 2. Analysis on Vibration Law of Transformer in Operation State

Transformer vibration can be divided into core vibration and winding vibration. Compared with the core vibration component, the winding component is extremely small, so this section mainly analyzes the core vibration law.

The magnetostriction of the core material *λ* will first increase to the maximum value max *λ*_max_. As the magnetic field intensity *H* increases, at this time, the magnetization *M* of the ferromagnetic material is the saturation magnetic domain wall shift magnetization *M*_WS_. The magnetostriction process at this stage is determined by the magnetic field. Domain wall shift dominates. When the magnetic field intensity *H* further increases, the magnetization *M* enters the saturation zone. The magnetostriction process at this stage is dominated by the magnetic domain rotation, while the magnetostriction gradually decreases (may be a negative value). In addition, according to the magnetostrictive experiment of soft magnetic materials [30], it is known that applying prepressure to the soft magnetic material in a specific direction can cause the magnetic domain wall to move (but will not affect the magnetic domain rotation), thereby affecting the magnetostrictive strain value. Thus, the transcendental function form of the iron core vibration characteristic *ε*_V_ in the normal state is shown in (1).

(1) $$\varepsilon_{V}=\frac{\lambda_{\max}-\lambda_{0}(\sigma )}{M_{\mathrm{MS}}^{2}}M^{2}$$

where the nonlinear function *λ*_0_(*σ*) is the magnetostriction value caused by the domain wall shift under the preload; the *λ*_max_ is the maximum magnetostriction value when the saturated wall moves. Similarly, the transcendental function form of the core vibration characteristics *ε*_V_ in DC bias state [31,32] is shown in (2).

(2) $${\varepsilon^{\prime }}_{V}=\frac{\lambda_{\max}-\lambda_{0}(\sigma {)}^{\prime }}{M_{\mathrm{MS}}^{2}}M+\frac{\lambda_{\max}-\lambda_{0}(\sigma {)}^{\prime }}{M_{\mathrm{MS}}^{2}}M^{2}+\frac{\lambda_{\max}-\lambda_{0}(\sigma {)}^{\prime \prime }}{M_{\mathrm{MS}}^{3}}M^{3}+\cdots$$

From (1) and (2), it can be seen that when the transformer is in normal state, *M* is the power frequency signal, and the main vibration frequency of transformer core is the double frequency of power frequency signal. Moreover, *M* is not a perfect sine wave in the actual operation process; it contains a small amount of high-order harmonic components, so the results of the experiment usually contain a small amount of *n* × 100 Hz (*n* = 2, 3, 4…). When DC bias occurs, *M* can be considered as the superposition of DC component and power frequency. At this time, the main vibration frequency of transformer core has components in *n* × 50 Hz (*n* = 1, 2, 3…), and the size of each component will increase with the increase of DC component in *M*.

## 3. Acoustic Signal Acquisition Experiment of Transformer in DC Bias State

### 3.1. DC Bias Experiment of 500 kV Transformer

In order to explore the characteristics of DC bias acoustic signal of an actual operation transformer, this paper collected the operation acoustic signal of the 500 kV Jingting substation before and after DC bias treatment. Before the treatment of DC bias, the neutral grounding value of the No. 2 transformer in Jingting station was 21.4 A, which exceeded the expected treatment standard (18 A), and the increase of transformer vibration and noise was obvious. After the DC bias treatment, the neutral grounding value decreased to 10.7 A, although there is still a certain degree of DC bias, but it has dropped to the governance standard.

**Figure 1:**
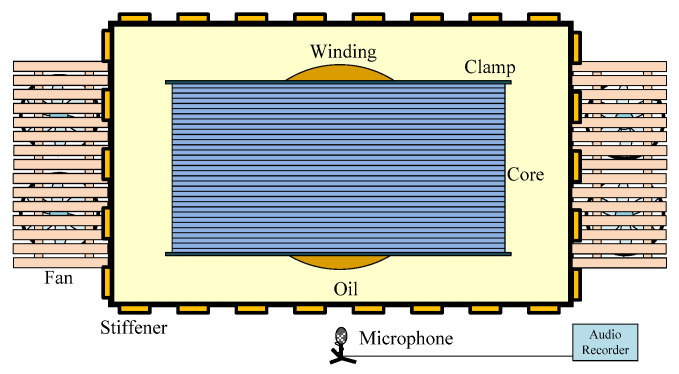
Schematic diagram of acoustic signal acquisition scheme for the transformer.

In the aspect of acoustic signal acquisition, according to IEC60651 standard, the measuring frequency band of the acquisition device covers 25 Hz~16 kHz, the sampling frequency is 96 kHz, and the frequency response meets the sampling requirements. The microphone should follow the following principles: (1) avoid the end face of the cooler; (2) be arranged on the side of the long end face; (3) be located in the middle of the end face; (4) the direction of the microphone is perpendicular to the position of the non-stiffener on the surface of the transformer; (5) the horizontal distance from the transformer tank wall is 100 cm; (6) the vertical distance from the ground is 150 cm. The schematic diagram of the acoustic signal acquisition scheme for the transformer is shown in Figure 1.

### 3.2. DC Bias Experiment of Core Model

**Figure 2:**
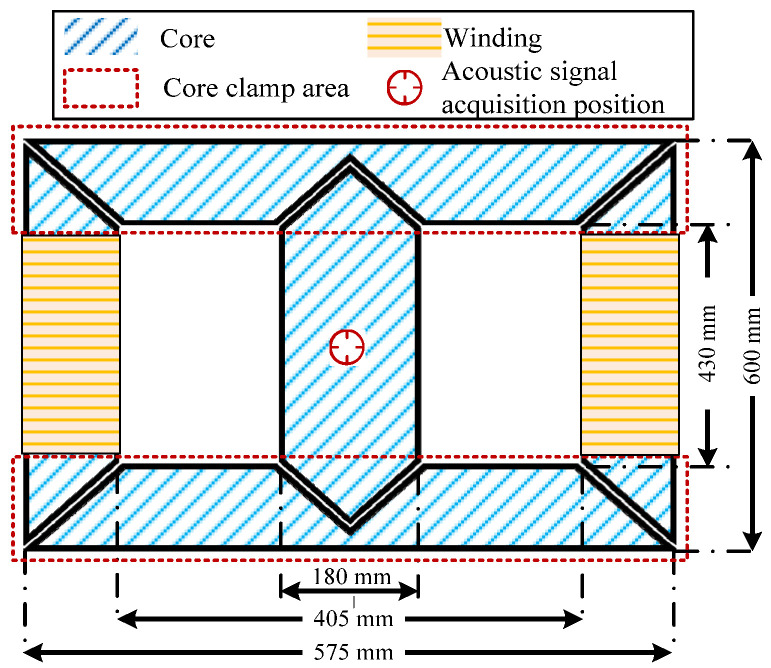
Model size and signal acquisition layout of the core.

**Figure 3:**
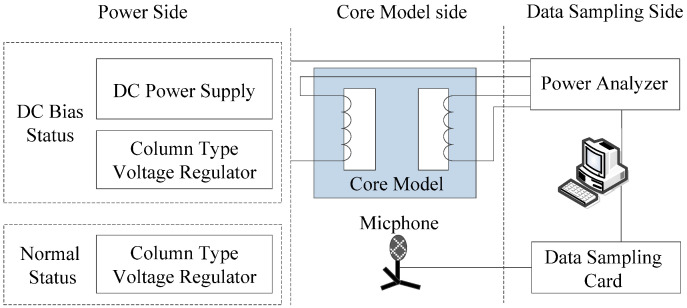
Experiment platform for the core model.

Most of the energy in the transformer acoustic signal is contributed by the core vibration. Therefore, a core model was made to simulate a transformer acoustic signal in the laboratory. The silicon steel sheet used in the model is B30P105, and its geometric dimension and signal acquisition layout are shown in Figure 2. In the aspect of acoustic acquisition, the same acquisition equipment as in Section 3.1 is used, which will not be repeated here. The excitation power supply equipment meets the test performance index of sinusoidal AC and DC bias. The whole core acoustic test platform is shown in Figure 3.

The magnetic flux density at the measuring point will directly affect the vibration and voiceprint of the iron core. Therefore, before applying different excitation signals, the test pressure level is determined according to the magnetic flux density amplitude of the core pillar to ensure that the iron core works in the unsaturated region. The pressurization level and scheme of the test are shown in Table 1.

## 4. Comparative Analysis of DC Bias Acoustic Signals

There are some differences in the acoustic parameters of DC bias between the real transformer and the laboratory core model. In order to find more suitable acoustic parameters for DC bias state early warning, it is necessary to compare and analyze parameters of the two groups of experiments.

### 4.1. Acoustic Signal Frequency Spectrum Analysis

**Figure 4:**
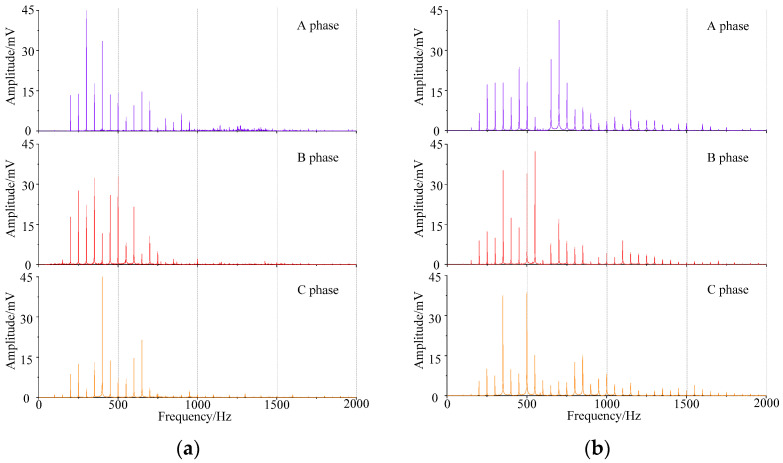
The 500 kV transformer acoustic signal frequency spectrum. (**a**) Normal state. (**b**) DC bias state.

The acoustic frequency domain signal of the 500 kV transformer in Jingting substation under normal state and DC bias state is shown in Figure 4. The acoustic frequency spectrum of the transformer in DC bias state has a large number aggregated at odd harmonics, part of the energy distribution above 700 Hz, and the fundamental frequency is very small. The dominant frequency of the three transformers is 500 Hz or above; under normal state, even harmonics such as 200 Hz, 300 Hz, and 400 Hz increase obviously, while even harmonics weaken, and the energy above 700 Hz weakens obviously, and the dominant frequency is distributed at 500 Hz and below.

**Figure 5:**
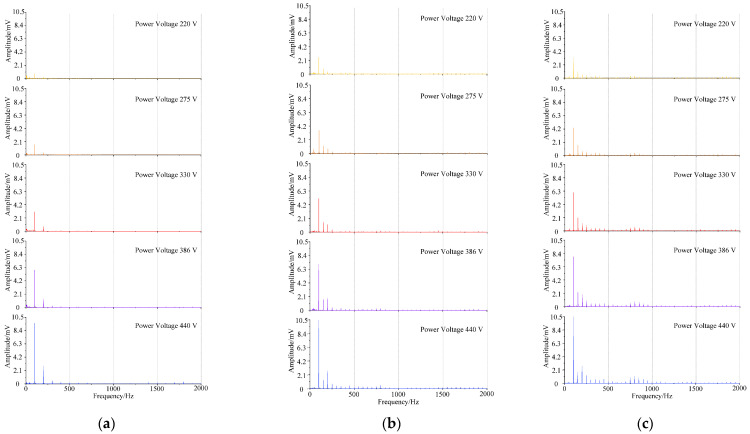
Transformer core model acoustic signal frequency spectrum. (**a**) Normal state. (**b**) DC bias current = 0.5 A. (**c**) DC bias current = 1.0 A.

The acoustic frequency domain signal of the core model under normal state and DC bias state is shown in Figure 5. The frequency spectrum is mainly distributed at integer multiples of 50 Hz, and the fundamental frequency (100 Hz) is the most important frequency. When the voltage rises, the amplitude of the transformer’s acoustic spectrum increases as a whole, and the proportion of each frequency in the total energy is almost unchanged; when the current of DC bias in the excitation of the transformer gradually increases, the proportion of the odd harmonics will increase, the high frequency component is gradually increasing, and the dominant frequency is stable at the fundamental frequency of 100 Hz.

### 4.2. Frequency Domain Parameters Comparative Analysis

According to the variation of acoustic signal in the above experiment, four typical parameters can be extracted:

(1) Dominant frequency *F*_1_,

(3) $$F_{1}=\arg\;\max(X_{f})$$

(2) Ratio between odd and even harmonics *F*_2_,

(4) $$F_{2}=\sum_{f=50}^{N-50}X_{f}/\sum_{f=100}^{N}X_{f}$$

(3) Ratio of fundamental frequency *F*_3_,

(5) $$F_{3}=X_{100}/\sum_{f=50}^{N}X_{f}$$

**Figure 6:**
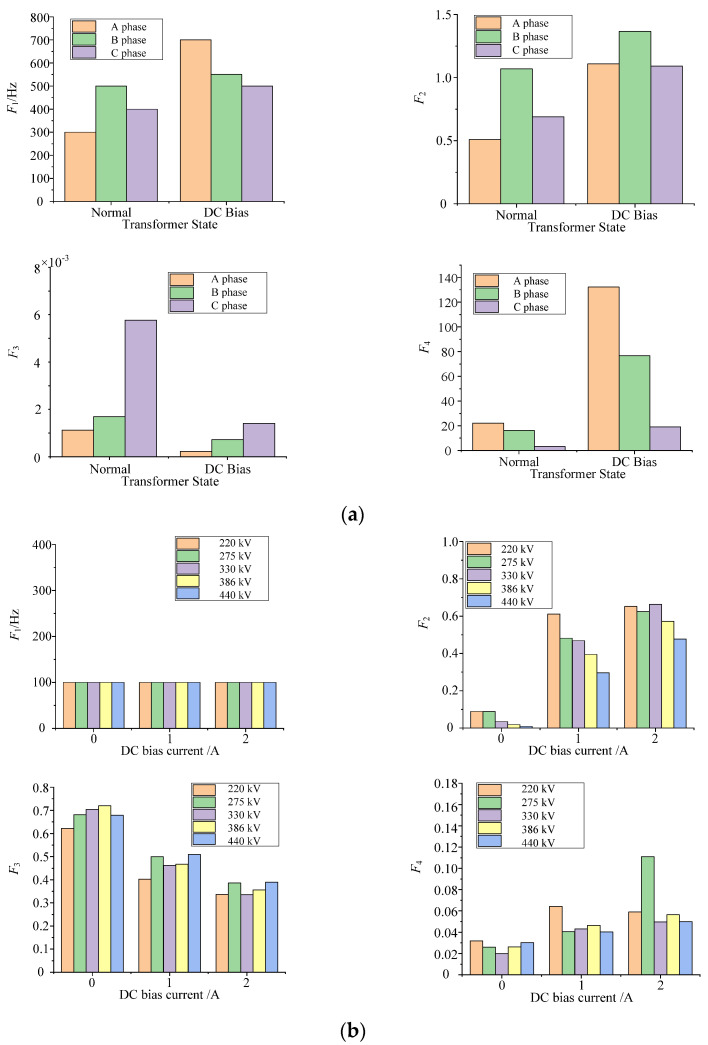
Variation of acoustic signal typical parameters of transformer DC bias. (**a**) 500 kV transformer. (**b**) Core model.

(4) Total harmonic distortion *F*_4_,
  (6) $$F_{4}=\sum_{f=f_{h}}^{N}X_{f}/\sum_{f=50}^{f_{h}}X_{f}$$
  where *X_f_* is the amplitude of frequency component at frequency *f*; *f*_max_ is the upper limit of statistical frequency. Because the energy of transformer acoustic signal is mainly distributed in the range of 0–2000 Hz, *f*_max_ is generally 2000 Hz. The calculation results are shown in Figure 6.

It can be seen from the calculation results of signal typical parameters that the actual case of the transformer parameters variation law is consistent with the core model, but there are some differences.

Consistency: Compared with the transformer acoustic signal in normal state, the actual case and the laboratory core model acoustic signal have the rule of fundamental frequency proportion decreasing, total harmonic distortion increasing, and ratio between 50 Hz and 100 Hz harmonic increasing, which is consistent with the theoretical analysis conclusion in Section 2.

Differences: (1) Compared with the transformer in normal state, the acoustic signal dominant frequency increased in the actual case of DC bias, but is always kept at 100 Hz in the laboratory core model. This is because there are many interference factors in the operation of the transformer, the voltage contains lots of high-frequency harmonics, and the DC current is large; the results show that the high-frequency harmonics of 50 Hz in acoustic signal share the energy of the fundamental frequency. (2) From the value of the parameter, there is a big gap between parameters from the laboratory core model and parameters from the actual transformer. For example, the ratio of the fundamental 500 kV transformer is in the range of 0.01% to 0.6%; however, that which is calculated by the core model is in the range of 35% to 75%. Other parameters have similar laws.

This paper holds that the difference in acoustic parameters between the core model and the real transformer is mainly caused by two reasons: (1) the DC bias current of transformer core model in the laboratory is small, so the frequency spectrum distortion of acoustic signal is weak; (2) the acoustic wave transmission medium of the real transformer is more complex, and the frequency response function of the oil and tank have an important impact on the acoustic frequency spectrum.

Based on the above two reasons, a frequency spectrum conversion model is proposed. The model is

(7) $$E_{R}(f)=D(f)\times H(f)\times E_{M}(f)$$

where *D*(*f*) is DC bias state frequency spectrum distortion function. *H*(*f*) is frequency response function of oil and tank. *E*_M_(*f*) is frequency spectrum of core model, and *E*_R_(*f*) is the simulated real transformer frequency spectrum.

In order to verify the effectiveness of the model, the acoustic signal of the core model (440 kV, 0.5 A) is calculated as a case.

**Figure 7:**
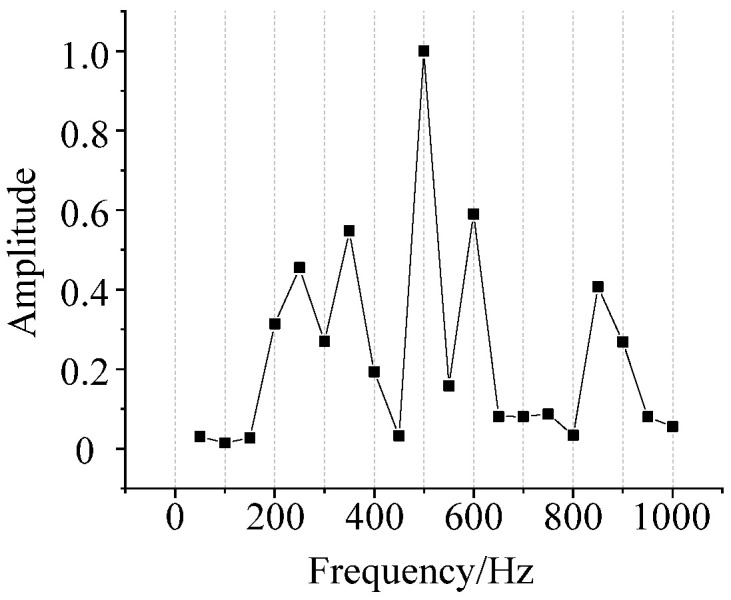
Frequency response function of oil and tank.

From the investigation work of [29], the frequency response function of the oil and tank *H*(*f*) is extracted. After normalizing, *H*(*f*) is shown in Figure 7. However, the upper limit of the frequency response function given in [29] is 1000 Hz. Therefore, only the amplitude of 0–1000 Hz of the spectrum can be calculated during the example verification.

**Figure 8:**
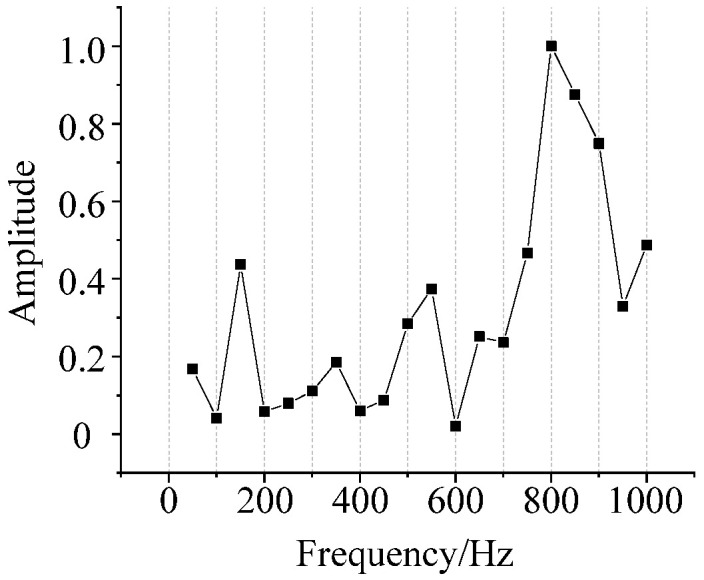
DC bias state frequency spectrum distortion function.

According to the frequency spectrum of 500 kV transformer under DC bias and normal state, *D(f)* can be preliminarily obtained. After averaging and normalizing the three phase transformers of data in Figure 4, the calculated *D(f)* is shown in Figure 8.

**Figure 9:**
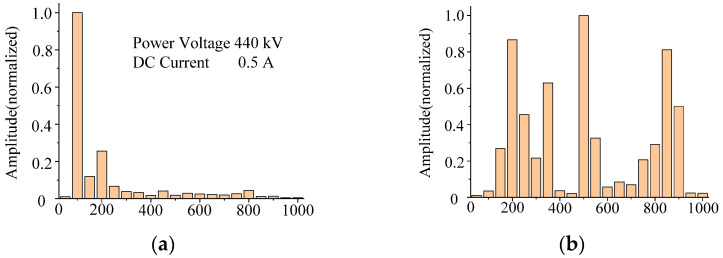
Comparison between the original spectrum and the calculated spectrum. (**a**) Core model frequency spectrum. (**b**) Simulated real transformer frequency spectrum.

The core model frequency spectrum *E*_M_(*f*) is shown in Figure 9a. A simulated real transformer frequency spectrum *E*_R_(*f*) can be calculated by *H*(*f*) given in Figure 6, *D(f)* given in Figure 7, and Formula (7).

According to Figure 9b, we can calculate four acoustic parameters mentioned in this paper. The results are shown in Table 2. Although the calculated high-frequency component may be too high, four items of the core model acoustic parameters are in the same range as the acoustic parameters of the real 500 kV transformer, and the order of magnitude of the two is very close. This result can prove that the conversion model is reasonable.

Therefore, the following conclusions can be drawn: (1) The reduction of ratio of fundamental frequency, the rise of total harmonic distortion, and the rise of ratio between odd and even harmonics are the necessary conditions to judge whether the transformer is in DC bias state, while the rise of dominant frequency is not a necessary condition. (2) The core model under the ideal condition and the actual case of the transformer can only compare a typical parameter variation rule, but the numerical comparison of parameters is meaningless.

The above two conclusions mean that the core model in the laboratory can provide some guidance for the selection of the actual transformer acoustic diagnosis parameters, but the results in the laboratory cannot provide data support for the identification of the anomalous state of the transformer. Therefore, we need an explicit acoustic diagnosis parameter warning threshold.

## 5. Determination of Acoustic Parameters Warning Threshold for DC Bias

According to conclusions of the previous chapter, the early warning method of the DC bias state is determined as follows:

Firstly, select parameters used for acoustic signal discrimination: three necessary discrimination parameters, including ratio between odd and even harmonics *F*_2_, ratio of fundamental frequency *F*_3_, and total harmonic distortion *F*_4_, are selected for early warning.

Then, determine the early warning threshold for the selected parameters: there are few anomalous cases on the real transformer. Although the laboratory model can produce a large amount of anomalous state acoustic signal data, according to conclusions of the previous chapter, the parameter value is not available in the real transformer warning work. Therefore, this paper collected a large number of normal transformer acoustic signal samples and made probability distribution statistics on the parameters of normal samples. Thus, the distribution of normal state parameters is obtained.

Finally, the upper or lower limit of the parameter distribution is used as the parameter early warning threshold. If the acoustic diagnostic parameters of a sample exceed the threshold, the sample is considered anomalous.

### 5.1. Acoustic Signal Acquisition of 162 Normal Transformers

In this paper, acoustic signal acquisition is carried out for 500 kV, 1000 kV transformers under the jurisdiction of State Grid Hebei Electric Power CO., LTD. Because all transformers are single-phase split transformers, and each group of transformers is relatively independent, this paper takes single-phase transformers as sample individuals for statistical analysis. The sample covers 162 transformers with 3 types of cooling methods, 10 manufacturers, and 21 stations. The sample library covers various types of transformers, which can support the reliability of acoustic signal parameter analysis results. The layout principle of microphone and acoustic signal acquisition equipment is the same as that in Section 3.1. The distribution of samples is shown in Table 3.

### 5.2. Acoustic Parameter Distribution

**Figure 10:**
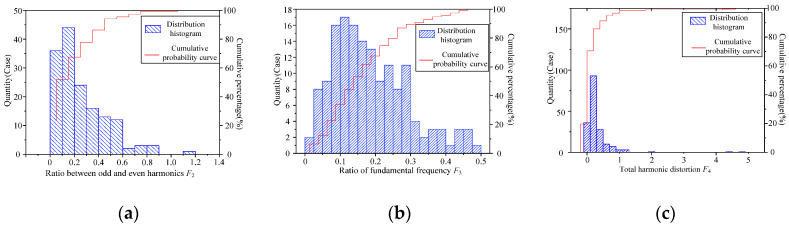
Distribution histogram and cumulative probability curve of transformer acoustic typical eigenvalues. (**a**) Ratio between odd and even harmonics *F*_2_. (**b**) Ratio of fundamental frequency *F*_3_. (**c**) Total harmonic distortion *F*_4_.

(1) The 50 Hz odd/even frequency multiplication ratio *F*_2_:

As shown in Figure 10a, the distribution histogram of *F*_2_ is shown. It can be seen from the figure that the *F*_2_ of transformer under normal state is mainly distributed between 0 and 0.6. According to the cumulative probability curve, 99% of the samples are below 0.9.

**Figure 11:**
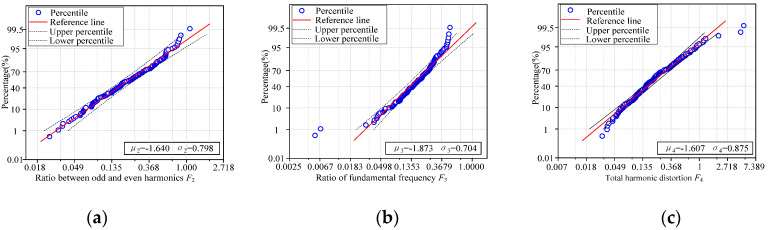
Lognormal probability plots of transformer acoustic typical eigenvalues. (**a**) Ratio between odd and even harmonics *F*_2_. (**b**) Ratio of fundamental frequency *F*_3_. (**c**) Total harmonic distortion *F*_4_.

The parameter distribution of *F*_2_ is right-skewed and presents the characteristics of lognormal distribution. *F*_2_ of the sample is ln*F*_2_ after calculating the logarithm, and ln*F*_2_ of all samples constitutes aggregate *D*_2_. Assume *D*_2_~N($\mu_{2}$,$\sigma_{2}$^2^), where $\mu_{2}$ is the mean value of ln*F*_2_ and $\sigma_{2}$ is the standard deviation of ln*F*_2_. The fitting results are $\mu_{2}$ = −1.640, $\sigma_{2}$ = 0.798, and the normal probability diagram is shown in Figure 11a. The data points in the figure are evenly arranged along the reference line, which verifies the rationality of the *F*_2_ obeying the lognormal distribution.

(2) Fundamental frequency proportion *F*_3_:

As shown in Figure 10b, the distribution histogram of *F*_3_ is shown. It can be seen from the figure that the *F*_3_ of transformer under normal state is mainly distributed between 0.025 and 0.3. According to the cumulative probability curve, 99% of the samples are above 0.025.

The parameter distribution of *F*_3_ is right-skewed and presents the characteristics of lognormal distribution. *F*_3_ of the sample is ln*F*_3_ after calculating the logarithm, and ln*F*_3_ of all samples constitutes aggregate *D*_3_. Assume *D*_3_~N($\mu_{3}$,$\sigma_{3}$^2^), where $\mu_{3}$ is the mean value of ln*F*_3_ and $\sigma_{3}$ is the standard deviation of ln*F*_3_. The fitting results are $\mu_{3}$ = −1.873, $\sigma_{3}$ = 0.704, and the normal probability diagram is shown in Figure 11b. The data points in the figure are evenly arranged along the reference line, which verifies the rationality of the *F*_3_ obeying the lognormal distribution.

(3) High/low-frequency ratio *F*_4_:

As shown in Figure 10c, the distribution histogram of *F*_4_ is shown. It can be seen from the figure that the *F*_4_ of the transformer under normal state is mainly distributed between 0.1 and 0.8. According to the cumulative probability curve, 99% of the samples are below 0.14.

The parameter distribution of *F*_4_ is right-skewed and presents the characteristics of lognormal distribution. *F*_4_ of the sample is ln*F*_4_ after calculating the logarithm, and ln*F*_4_ of all samples constitutes aggregate *D*_4_. Assume *D*_4_~N($\mu_{4}$,$\sigma_{4}$^2^), where $\mu_{4}$ is the mean value of ln*F*_4_ and $\sigma_{4}$ is the standard deviation of ln*F*_4_. The fitting results are $\mu_{4}$ = −3.074, $\sigma_{4}$ = 0.520, and the normal probability diagram is shown in Figure 11c. The data points in the figure are evenly arranged along the reference line, which verifies the rationality of the *F*_3_ obeying the lognormal distribution.

### 5.3. Early Warning Threshold and Example Verification

According to the rule obtained from the experiment in Section 4.2, the DC bias state of the transformer will cause *F*_2_ and *F*_4_ of the acoustic signal to rise, and *F*_3_ to fall. Thus, after excluding a small number of outliers, the 99% quantile line was selected as warning threshold of *F*_2_ and *F*_4_, and the 1% quantile line was selected as warning threshold of *F*_3_.

**Figure 12:**
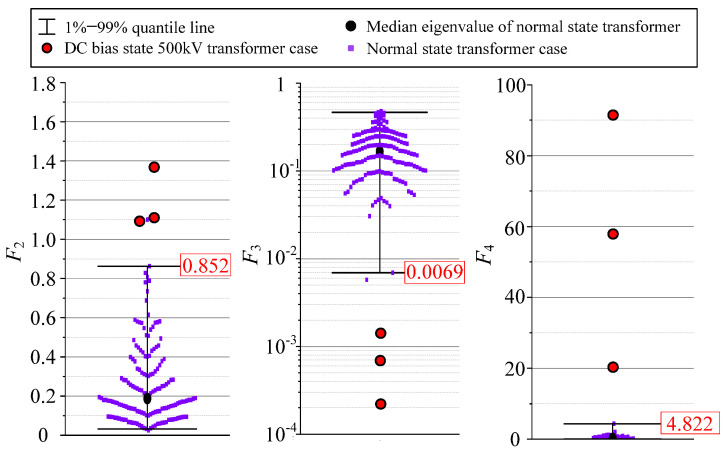
Comparison of typical parameters warning threshold and anomalous case.

In order to test the effectiveness of the warning threshold set above, in this paper, the DC bias state 500 kV transformer cases in Section 3.1 are compared to the parameters warning threshold, and comparison results are shown in Figure 12: *F*_2_, *F*_3_, and *F*_4_ of DC bias state 500 kV transformer cases all exceed the warning threshold. The comparison results show that the early warning threshold given according to the statistics of three parameters can identify the DC bias state.

## 6. Conclusions

In this paper, by comparing the acoustical parameters of the core model with the 500 kV transformer, three typical parameters were selected for DC bias state acoustic warning, and clear parameter warning thresholds were given.

(1) The dominant frequency *F*_1_ variation law of acoustic signal in the laboratory core model is different from that in the 500 kV real transformer. This is because the DC bias state of the real transformer is more serious, and the acoustic signal of the real transformer will be affected by the frequency response function of the acoustic transmission medium.
(2) Under ideal laboratory conditions, the core model and DC bias state acoustic signal of field transformer can only compare their acoustical parameters with state changes, but cannot compare the values with each other. Therefore, the data of the laboratory model cannot be used to delineate the parameters warning threshold.
(3) The numerical distribution of *F*_2_, *F*_3_, and *F*_4_ parameters of a large number of transformer acoustic samples in the normal state has strong aggregation. The comparison results of real cases illustrate that the warning threshold given in this paper based on three-item parameter data statistics enables stable identification of DC bias state.

## Figures and Tables

**Table 1:** Voltage levels and excitation plans for test.

Status	Voltage	DC Bias	Magnetic Flux Density of Core Center Pillar
Normal	193 V	0 A	0.7 T
	276 V	0 A	1.0 T
	330 V	0 A	1.2 T
	386 V	0 A	1.4 T
	440 V	0 A	1.6 T
DC bias	193 V	0.5 A	-
	276 V	0.5 A	-
	330 V	0.5 A	-
	386 V	0.5 A	-
	440 V	0.5 A	-
	193 V	1.0 A	-
	276 V	1.0 A	-
	330 V	1.0 A	-
	386 V	1.0 A	-
	440 V	1.0 A	-

**Table 2:** Comparison of acoustic parameters calculation results.

Parameters	Parameter Values		
	Value of 500 kV Transformer	Value of Core Model	Value of Core Model (Calculated with Frequency Spectrum Conversion Model)
*F*_1_	>300 Hz	100 Hz	500 Hz
*F*_2_	>0.5	0~0.65	0.972
*F*_3_	<0.0058	0.336~0.720	0.0054
*F*_4_	>3.31	0.019~0.111	3526.836

**Table 3:** Substation of transformer in sample library.

Substation Name	Voltage Level/kV	Quantity/Set	Substation Name	Voltage Level/kV	Quantity/Set
Xingtai	1000	6	Baoding	1000	6
Xinji	500	12	Qingyuan	500	12
Pengcun	500	12	Shibei	500	9
Lianzhou	500	9	Xinan	500	9
Linhe	500	9	Cangxi	500	9
Ciyun	500	9	Xingxi	500	6
Zongzhou	500	6	Wuyi	500	6
Huanghua	500	6	Guishan	500	6
Guangyuan	500	6	Yishui	500	6
Yingzhou	500	6	Yuanshi	500	6
Xuanhuihe	500	6			

## Data Availability

The data that support the findings of this study are available from the corresponding author upon reasonable request.
